# Prevalence of Diabetes in Nepali-Speaking Bhutanese Americans Living in the Greater Harrisburg Area

**DOI:** 10.7759/cureus.39698

**Published:** 2023-05-30

**Authors:** Bishal Kadariya, Sulabh Neupane, Andrew J Wakeling, Nishta R Polam, Meghan L Wilson

**Affiliations:** 1 Medical School, Edward Via College of Osteopathic Medicine, Blacksburg, USA; 2 Faculty for Nutrition, Cell Biology, and Physiology, Edward Via College of Osteopathic Medicine, Blacksburg, USA

**Keywords:** physical activity, rice consumption, nepali-speaking bhutanese, refugees, bhutanese-nepali, diabetes

## Abstract

Members of the Nepali-speaking Bhutanese refugee community had resettled in the United States beginning in 2008 after previously being settled in United Nations (UN) refugee camps in Nepal. Due to the recency of their resettlement, there has been little research regarding diabetes in the Nepali-speaking Bhutanese American community. This study sought to identify the prevalence of diabetes in Nepali-speaking Bhutanese Americans living in the Greater Harrisburg Area and whether this community was at a higher risk of developing diabetes due to changes in diet and physical activity lifestyle behaviors. This study was conducted using an anonymous online survey. Anyone over the age of 18 and a self-identified member of the Nepali-speaking Bhutanese American community living in the Greater Harrisburg Area was included, regardless of their diabetes status. This study excluded individuals under the age of 18, those found outside the limits of the targeted region, and those who do not self-identify as members of the Nepali-speaking Bhutanese American community. Through this survey, data regarding demographics (age and gender), length of stay in the US, diabetes status (present or absent), consumption of rice (increased or decreased post-resettlement), and physical activity status (increased or decreased post-resettlement) were collected. The current prevalence of diabetes in this population was compared against the one reported by the CDC before migration and against the prevalence of diabetes in the general population of the United States of America (USA). The association between rice consumption, physical activity, and diabetes was analyzed using the odds ratio. The survey yielded responses from 81 participants. Results showed a 2.29 times higher prevalence of diabetes in the Bhutanese-speaking Nepali population of the Greater Harrisburg Area, Pennsylvania, compared to the general population of the USA. Results indicated a 37 times higher prevalence of diabetes after resettlement in the USA compared to the population’s self-reported prevalence before the resettlement. The data showed that increased rice consumption or decreased physical activity alone did not significantly increase the risk of developing diabetes. However, the combination of decreased physical activity and increased rice consumption significantly increased the risk of diabetes, with an odds ratio of 5.94 (CI: 1.27 to 27.56, p-value: 0.01). The higher prevalence of diabetes in this community justifies diabetes education around causes, symptoms, treatments, and preventative healthcare methods. Greater awareness of the issue among the members of this community, as well as their healthcare providers, paves the way for future studies to identify all possible risk factors for diabetes in this community. Once risk factors are identified, early interventions and screening tools can be implemented to mitigate the onset of disease in this population in the future.

## Introduction

Diabetes 

Over 37 million Americans of all ages have diabetes, and its prevalence continues to rise [1]. In non-Hispanic Asian adults aged 18 years or older, the prevalence of diabetes was 9.5%, with Asian Indians having the highest prevalence [1]. Diabetes is a disease of decreased insulin production by the pancreas (Type one diabetes) or insulin resistance (Type two diabetes), leading to high blood glucose and its associated symptoms, such as dry mouth, frequent fatigue, and decreased vision [2]. Highly processed foods and decreased levels of physical activity are associated with an increased prevalence of diabetes. Both genetic and metabolic factors are additional causes of this rise [3]. Risk factors such as family history, age, and ethnicity are categorized as non-modifiable risk factors, whereas smoking, obesity, physical inactivity, and an unhealthy diet can be modified [3].

Due to a wide range of dietary practices around the globe, foods associated with diabetes are variable [4]. The “energy-dense Western-style” diet, like processed foods and convenience foods, along with inadequate physical activity, are considered the culprits for the high prevalence of diabetes in Western countries [4]. In contrast, rice is a staple food source in Nepal and meets more than 50% of grain and 30% of caloric requirements [5]. In Asian countries, rice has been linked with an increased risk of diabetes [6,7]. Rice has a higher glycemic index than whole grains, causing a more rapid spike in blood glucose levels, thus increasing the risk for type 2 diabetes [7]. Studies have shown that rice consumption is associated with the development of type 2 diabetes, and the risk is higher in Asian populations compared to Western populations [6,7]. A meta-analysis on white rice consumption and the risk of type 2 diabetes found that a single serving of rice per day increased the risk of type 2 diabetes by 11% in the general population [7]. These studies support the association between rice consumption and diabetes.

All types of physical activities, including occupational and leisure time physical activity, resistance exercises, and walking, were found to be associated with a decreased risk for type 2 diabetes [4,8,9]. Among adults 18 or older diagnosed with diabetes in the United States of America (USA), 38% reported physical inactivity, which was defined as less than 10 minutes of moderate or vigorous activity per week [1]. A study (n=20,757) showed that physically active men had a reduced risk (by 20%) for diabetes regardless of their body mass index (BMI) compared to physically inactive men [9]. Conversely, sedentary time (e.g., watching TV, using the computer, being seated while communicating, or at the workplace) has been associated with an increased risk for type 2 diabetes [4,10]. Another study (n=3,232) reported an approximate 3.5% increase in the risk of diabetes with each hour spent watching TV in 3.2 years [10]. 

Refugee health and diabetes

The United States has received more than three million refugees since 1975 [11]. A refugee is a person fleeing to find safety in another country due to war, violence, conflict, or persecution in one’s home country [12]. While the United Nations High Commissioner for Refugees (UNHCR) searches for a relocation country, the refugees are kept in refugee camps, where they are faced with navigating and overcoming tremendous hardships, including food insecurity, relocation uncertainty, a lack of healthcare access, prejudice, language, and cultural barriers [13,14]. Once relocated to a new country, refugees must adjust to several new environments and cultures, which can pose challenges to accessing cultural, social, and economic capital and can lead to social isolation [15]. Owing to the scarcity of basic needs, refugees frequently lack proper screenings for diseases and immunizations, and their health can worsen by the time care is established due to barriers to accessing healthcare [11].

The prevalence of diseases such as diabetes, obesity, and dyslipidemia tends to be higher in various refugee populations in the USA [16]. A previous study (n=1,156) suggests that the prevalence of diabetes among Somali refugees was higher than the general USA population (21.2% vs. 15.9% in men and 14.6% vs. 12.6% in women) [17]. A review of multiple studies looking at the health outcomes of resettled refugees in the USA reported that refugees have an increased risk of developing diabetes compared to USA-born controls and a 12% increase in the odds of developing diabetes per year in the USA post-resettlement [18]. In the same review, the prevalence of diabetes in all studied refugees ranged from 5% to 6%, with 9% to 17% in African refugees, 6% to 14% in Bhutanese refugees, and 6% to 16% in Iraqi refugees [18].

Along with the prevalence of disease, refugee populations have lower rates (30% in women and 39% in men) of physical activity than the general population (43% in women and 46% in men) [16,17]. Men and women of the Somali refugee community in Minneapolis and St. Paul, Minnesota, are less physically active than the general US population [17]. In a study (n=100) with the Bhutanese refugees in Houston, only 41% of the subjects reported participating in at least 20-30 minutes of aerobic exercise daily [19].

Diet plays a crucial role in the development of diabetes, especially a high-carbohydrate diet. Rice consumption has been shown to have a positive correlation with the development of diabetes [20]. The study [19] of the Bhutanese refugee population in Houston further looked at the community members’ dietary behaviors. While rice consumption was not reported in this study (n=100), 62% of the participants reported consuming fruits and vegetables in the recommended proportions, and 47% reported consuming fried foods a few times a week [19]. A study [20] found a positive correlation between the consumption of rice and digestive diseases. 

Nepali-speaking Bhutanese refugees

Nearly 100,000 Nepali-speaking Bhutanese (NSB) were displaced from their homes between 1990 and 1993 [21,22]. Divided into seven camps, they spent nearly two decades in eastern Nepal as refugees [23]. The World Food Program (WFP) and UNHCR distributed food rations, including rice, lentils, chickpeas, vegetable oil, sugar, salt, and fresh vegetables, to these refugees. Additional food, including meat, was available for purchase at markets outside the camps but was costly. Hence, rice was the most commonly consumed food in the camps [22]. Despite being a staple food, rice consumption was limited due to rationing. 

Starting in 2008, NSB refugees were granted the opportunity to resettle in eight different countries, including the USA, Canada, and Australia [22,23]. The USA accepted most NSB refugees, with about 96,000 people resettling in the USA by 2019 [14,15,23]. Pennsylvania is among the top 10 states to host these refugees, with the Harrisburg-Carlisle metropolitan area containing a dense population [22,24].

The Centers for Disease Control reports the pre-migration self-reported prevalence of diabetes as 0.7% [22]. Being a rather new population in the USA, it is vital to identify the prevalence of diabetes in NSB Americans. It is equally vital to identify associated lifestyle behaviors that could lead to the prevalence of this disease. The purpose of this study was to identify and compare the prevalence of diabetes in NSB Americans to the general US population. This study also aims to compare the current prevalence of diabetes in this community to the prevalence before resettlement. In addition, the study intended to examine the relationship between diabetes, rice consumption, and physical activity. Realizing the prevalence and understanding the relationship between diabetes and the previously mentioned factors could help healthcare providers deliver culturally competent care to these patients. 

This article was previously presented as a research abstract poster at the 2023 Edward Via College of Osteopathic Medicine Research Day on February 24, 2023. 

## Materials and methods

A cross-sectional survey study was designed to assess the demographics and self-reported prevalence of diabetes in the target population. The target population is defined as NSB Americans living in the Greater Harrisburg Area (GHA). For this study, the GHA was defined by the Cumberland County, Dauphin County, Perry County, and Lebanon County borders in Pennsylvania. This study received ethical approval from the Edward Via College of Osteopathic Medicine Institutional Review Board.

**Figure 1:**
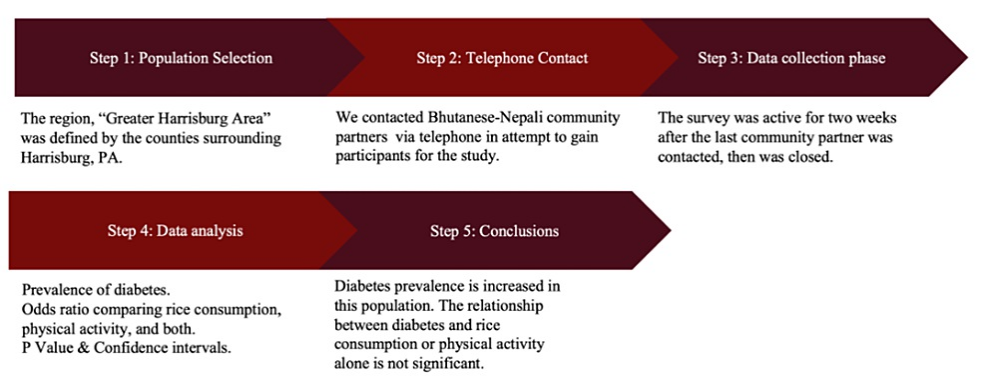
Flowchart illustrating the data collection and interpretation processes utilized in the study

To recruit participants, a script was designed to reach out to local businesses, temples, and community organizations to ask if they would be willing to post the informational flier and link to the survey (Figure 1).

Contact was made through the participants’ publicly available phone numbers or social media pages. Strict limits on the number of times an entity could be contacted were set at three attempts. If an entity declined to participate in the recruitment drive, they were not re-contacted. Records of the contact attempts and disposition towards participation were recorded in a spreadsheet to ensure adherence. Once entities accepted to participate in recruitment, QR code links were posted in the physical location of the business, temple, or organization and/or on their social media page for Nepali patrons to access of their own volition. Information about the study and survey was provided before beginning the survey. This information included the fact that consent was implied by the submission of the survey. Individuals recruited for the study were not compensated for volunteering to participate.

Anyone over the age of 18 who self-identified as a member of the NSB American community living in GHA was included, regardless of their diabetes status. This study excluded individuals under the age of 18, those found outside the limits of the targeted region, and those who do not self-identify as members of the NSB American community. The survey was designed to be a minimal risk for participants. In addition to the English version, a Nepali-translated script and voice recording for each question were included in the survey for participants who may have limited reading comprehension. However, this survey was not validated before administration. The written translation was done by a native speaker who works as a medical translator. The voice recording was of a native speaker. The questionnaire (Appendix A) asked basic demographic questions such as age and gender, length of stay in the USA, whether they have been diagnosed with diabetes, and if yes, for how long, and lastly, whether their rice consumption and physical activity levels have increased or decreased after resettlement. 

The survey period concluded two weeks after the end of the recruitment phase. Following the conclusion of the survey, the results were exported and analyzed in this cross-sectional study. Survey responses that were complete were used in data analysis, and incomplete responses were excluded from the study. The strength of association for increased rice consumption and decreased physical activity, and both increased rice consumption and decreased activity related to those with and without diabetes, was calculated using the odds ratio. For each value, the confidence interval and p-value were calculated. A confidence interval of 95% was considered significant, and a p-value of < 0.05 was considered significant.

## Results

The survey yielded 81 complete and 15 incomplete responses from participants through this recruitment drive (Table 1).

**Table 1:** Survey data is represented here as a count of survey responses categorized by those with or without diabetes. The percent calculated is the quotient of the survey response count and the total number of respondents with diabetes (n1) or the total number of respondents without diabetes (n2). US: United States

		With Diabetes (n1=21)	Without diabetes (n2=60)
Sex			
	Male	8 (38.10%)	33 (55%)
	Female	13 (61.90%)	27 (45%)
Age			
	18-40	8 (38.10%)	43 (71.67%)
	40-65	5 (23.81%)	13 (21.67%)
	> 65 years	8 (38.10%)	4 (6.66%)
Length of time in the US			
	0-5 years	1 (4.76%)	1 (1.66%)
	> 5 years	19 (90.48%)	59 (98.33%)
	No response	1 (4.76%)	0 (0%)
Rice consumption			
	Increased	8 (38.10%)	12 (20%)
	Decreased	9 (42.86%)	26 (43.33%)
	No change	4 (19.05%)	22 (36.67%)
Physical Activity			
	Increased	0 (0%)	10 (16.67%)
	Decreased	14 (66.67%)	29 (48.33%)
	No change	6 (28.57%)	20 (33.33%)
	No response	1 (4.76%)	1 (1.67%)

Of the 81 participants, 49.38% were females (n=40), and 50.62% were males (n=41). Of the respondents, 62.96% were between the ages of 18 and 40 (n=51), 22.22% were between ages 41 and 65 (n=18), and 14.81% were over the age of 65 (n=12). 96.30% of survey participants reported having lived in the USA for more than five years (n=78), 2.47% reported having lived in the USA for less than five years (n=2), and 1.23% did not wish to answer the question (n=1). 

Pertaining to diabetes and lifestyle behaviors, 25.93% reported having diabetes (n=21), and 74.07% responded they did not have diabetes (n=60). Of the 21 respondents who reported having diabetes, 38.10% were male (n=8), and 61.90% were female (n=13). 38.10% of them were aged 65 or older (n=8), 23.81% were between the ages of 41 and 65 (n=5), and 38.10% were between the ages of 18 and 40 (n=8). 61.90% of them had been diagnosed more than five years ago (n=13), 28.57% were diagnosed in the last five years (n=6), and 9.52% did not answer the question (n=2). 4.76% had been living in the USA for less than five years (n=1), 90.48% for more than five years (n=19), and 4.76% did not wish to answer the question (n=1). 

Rice consumption increased for 24.69% of respondents (n=20), decreased for 43.21% of respondents (n=35), and stayed the same for 32.1% of respondents (n=26). Exercise increased for 12.35% of respondents (n=10), decreased for 53.09% of respondents (n=43), stayed the same for 32.1% of respondents (n=26), and 2.47% of respondents did not wish to answer the question (n=2). Of the respondents who reported having diabetes, 38.10% had increased rice consumption (n=8), 66.66% reported decreased physical activity (n=14), and 23.81% reported both increased rice consumption and decreased physical activity (n=3). 

Of the participants who did not have diabetes, 28.33% reported increased rice consumption (n=17), 48.33% reported a decrease in physical activity (n=29), and 5.00% reported having both increased rice consumption and decreased physical activity. The odds ratio of having diabetes with increased rice consumption was 1.56 (CI: 0.54 to 4.42, p-value: 0.203); with decreased physical activity, it was 2.14 (CI: 0.76 to 6.04, p-value: 0.075); and with increased rice consumption and decreased physical activity combined, it was 5.94 (CI: 1.27 to 27.56, p-value: 0.01). 

## Discussion

Diabetes is a significant source of morbidity and healthcare costs in the USA. It is estimated to cost $327 billion to the US economy as of 2017 [25]. Indirectly, diabetes causes decreased productivity and increased absenteeism at the workplace, adding up to $26.9 billion and $3.3 billion, respectively [25]. Identifying populations that are at increased risk can reduce morbidity and the associated strain on the healthcare system through earlier detection [25]. This study identified that the NSB American population has a 2.29 times higher prevalence than the general population of the USA (Table 2), indicating that the population could benefit from enhanced diabetes screening and education on ways to prevent developing diabetes.

**Table 2:** Compares the prevalence found in this study with the prevalence reported by prior studies. CDC: Centers for Disease Control and Prevention [1,18,22]

	Reported Prevalence
Prior to Resettlement (Refugee Health Profile, CDC) [22]	0.7%
Prior Studies after Resettlement (2008 to 2016) [18]	6-14%
General Population in the USA (per CDC) [1]	11.3%
This study (2022)	25.9%

This study identified that the NSB American population has a diabetes prevalence 37 times higher than before resettlement in the USA (Table 2).

However, due to the lack of diagnostic means in refugee camps, there is a possibility for a higher than-reported prevalence before migration. Prior studies [18] have identified the prevalence of diabetes as 6%-14% in the Bhutanese refugee population (table two). These studies [18] took place early after resettlement, between 2008 and 2016. The prevalence identified in this study was 1.85 to 4.32 times higher than in these prior studies. Roughly 90% of respondents in this study have been living in the USA for more than five years. This increase in prevalence is speculated to be caused by increased exposure time. 

This study speculated that after resettling in the US, rice consumption would have increased, and physical activities would have decreased. Carbohydrate consumption is positively correlated, and physical activity is negatively correlated with the development of diabetes [8,10,20]. Results show that rice consumption increased for nearly one-fourth, and physical activity decreased for more than half of the study participants. When examined individually, self-reported rice consumption and decreased physical activity did not show a significant increase in the risk of developing diabetes. However, the combination of decreased exercise and increased rice consumption was significantly associated with diabetes (Table 3). 

**Table 3:** Odds of developing diabetes with increased rice consumption and decreased physical activity. RC: rice consumption, PA: physical activity, CI: confidence interval.

	OR (95% CI)	p-value
Diabetes and Increased RC	1.56 (0.55 - 4.42)	0.20%
Diabetes and Decreased PA	2.14 (0.76 - 6.04)	0.08%
Diabetes and Increased RC and Decreased PA	5.94 (1.28 - 27.56)	0.01%

Limitations

This study used convenience sampling because random sampling was not practical. Recall bias and self-report bias were limitations in the study design. There was also no way to quantify the number of participants who had undiagnosed diabetes prior to immigration. Diabetes has many factors that influence its development, but this study only measured two variables: rice consumption and physical activity. The question about rice consumption did not include other sources of carbohydrates that could possibly contribute to the results. **It should be realized that the NSB American population in GHA is an isolated group, limiting the sample size.** Other refugee communities have different backgrounds and are potentially subjected to different conditions. Thus, findings from this study might be limited, and hence the same conclusions cannot be inferred from other communities. 

Future research

Studies specifically targeted at breaking down each individual contributor to diabetes should follow. Enhanced knowledge of such health changes, their causes, and their effects would facilitate the development of appropriate screening measures for diabetes. Future research should better characterize the change in diet among this population by including a more comprehensive measurement of carbohydrate consumption and the inclusion of other foods contributing to the diagnosis of diabetes (e.g., processed foods, sugar-sweetened beverages, baked goods, etc.). Additional measures can be utilized to determine daily physical activity engagement in this population. A qualitative study on how participants' lifestyles have changed since immigration could be used to uncover additional lifestyle behaviors and other health conditions contributing to the increased prevalence of diabetes. Studies examining the trends in the prevalence of diabetes after resettlement should follow in order to identify any correlations between years spent in the US and the diagnosis of diabetes. Having access to the specific determinants of their health allows community members to be proactive in implicating preventative measures.

## Conclusions

This study is among the few to research diabetes in this population. The findings of this study suggest that the study population is at a relatively higher risk for the development of diabetes. Given the findings of this study, healthcare providers, public health workers, community leaders, and other parties of interest should work on implementing measures to inform community members of this risk. Preventative measures should be established, and community members should be educated on risk factors, including dietary and other lifestyle factors, in addition to the pathophysiology and management of diabetes.

## Appendices

Appendix A

The survey questions are represented here. English followed by a Nepali translation was used for ease of use by participants who may have varying levels of proficiency in reading English. An audio recording of a native speaker reading the Nepali translation was embedded in each question.

1. What is your biological sex? (तपाईको जैविक लिङ्ग के हो?)

 a. Female (महिला)

 b. Male (पुरुष)

 c. Do not wish to answer (म जवाफ दिन चाहन्न)

2. What is your age range? (तपाईंको उमेर दायरा के हो?) 

 a. 18-40 years (१८-४० वर्ष)

 b. 41- 65 years (४१ - ६५ वर्ष)

 c. Above 65 years (६५ वर्ष माथि)

 d. Do not wish to answer (म जवाफ दिन चाहन्न)

3. How long have you been in the US? (तपाई अमेरिकामा बसेको कति भयो?)

 a. 0-5 years (०-५ वर्ष)

 b. > 5 years (५ वर्ष माथि)

 c. Do not wish to answer (म जवाफ दिन चाहन्न)

4. Have you ever been told by a doctor that you have diabetes, or have you ever been diagnosed with type II diabetes? (के तपाईलाई कहिल्यै डाक्टरले मधुमेह/ चिनी रोग भएको बताउनुभएको छ वा तपाईलाई मधुमेह / चिनी रोग भएको निदान गरिएको छ?)

a. Yes (छ)

b. No (छैन)

c. Do not wish to answer (म जवाफ दिन चाहन्न)

5. If you answered ‘Yes’ to question 4, how long has it been since you have been diagnosed with diabetes? (यदि तपाईंले प्रश्न 4 को 'छ' जवाफ दिनुभयो भने, तपाईलाई मधुमेह / चिनी रोग भएको कति समय भयो?)

a. 0-5 years (०-५ वर्ष)

b. 5 years or more (५ वर्ष माथि)

c. Not Applicable (लागु हुँदैन)

d. Do not wish to answer (म जवाफ दिन चाहन्न)

6. Has your consumption of rice increased or decreased after moving to the US? (अमेरिका आएपछि के तपाईको भातको खपत बढेको छ कि घटेको छ?)

a. Increased (बढेको छ)

b. Decreased (घटेको छ)

c. Stayed the same (उस्तै छ)

d. Do not wish to answer (म जवाफ दिन चाहन्न)

7. Has your physical activity increased, decreased, or stayed the same after moving to the US? (अमेरिका आएपछि तपाईको शारीरिक गतिविधि बढ्यो, घट्यो वा उस्तै छ?)

a. Increased (बढेको छ)

b. Decreased (घटेको छ)

c. Stayed the same (उस्तै छ)

d. Do not wish to answer (म जवाफ दिन चाहन्न)
